# High performance CHO cell line development platform for enhanced production of recombinant proteins including difficult-to-express proteins

**DOI:** 10.1186/1753-6561-7-S6-P75

**Published:** 2013-12-04

**Authors:** Pierre-Alain Girod, Valérie Le Fourn, David Calabrese, Alexandre Regamey, Deborah Ley, Nicolas Mermod

**Affiliations:** 1 Selexis SA, Plan-Les-Ouates, Switzerland; 2 University of Lausanne, Switzerland

## Background

In an effort to improve product yield of mammalian cell lines, we have previously demonstrated that our proprietary DNA elements, Selexis Genetic Elements (SGEs), increase the transcription of a given transgene, thus boosting the overall expression of a therapeutic protein drug in mammalian cells [1]. However, there are additional cellular bottlenecks, notably in the molecular machineries of the secretory pathways. Most importantly, mammalian cells have some limitations in their intrinsic capacity to manage high level of protein synthesis as well as folding recombinant proteins. These bottlenecks often lead to increased cellular stress and, therefore, low production rates.

## Material and Methods

Our specific approach involves CHO cell line engineering. We constructed CHO-M libraries based upon the CHO-M genome and transcriptome and using unique proprietary transposon vectors harboring SGE DNA elements to compensate for rate-limiting factors [2]. Each CHO-M*plus *library displays a diversity of auxiliary proteins involved in secretory pathway machineries and cellular metabolism. Collectively, the libraries address a broad range of expression issues.

## Results

**Figure 1:**
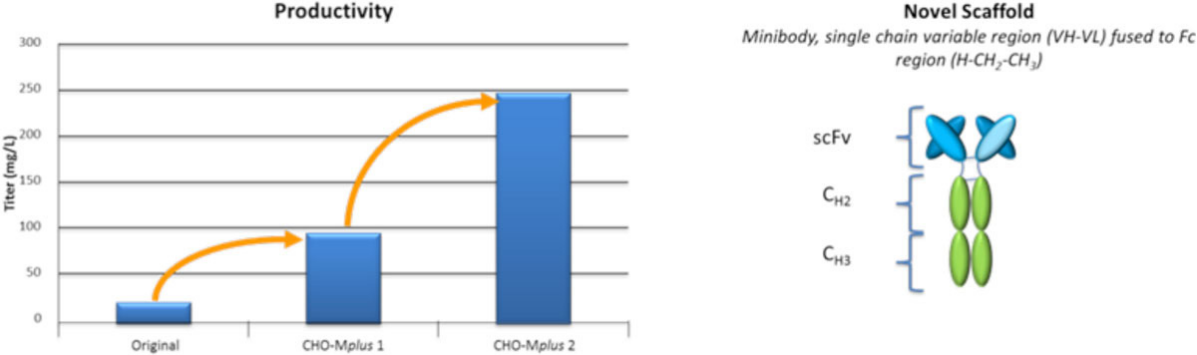
**The iterative application of the CHO-Mplus libraries enabled >10 fold increase in productivity of ScFv:Fc without changes in gene copy number or transcription level of gene of interest**.

Figure 1 shows that our CHO-M*plus *libraries enabled the selection of a clonal cell line expressing 12 fold more product by comparison to the unmodified host cell [3].

## Conclusions

Our results demonstrate that components of the secretory and processing pathways can be limiting, and that engineering of the metabolic pathway ('omic' profiling) improves the secretion efficiency of therapeutic proteins from CHO cells.
